# Early Impairment of Lung Mechanics in a Murine Model of Marfan Syndrome

**DOI:** 10.1371/journal.pone.0152124

**Published:** 2016-03-22

**Authors:** Juan J. Uriarte, Thayna Meirelles, Darya Gorbenko del Blanco, Paula N. Nonaka, Noelia Campillo, Elisabet Sarri, Daniel Navajas, Gustavo Egea, Ramon Farré

**Affiliations:** 1 Unitat Biofísica i Bioenginyeria, Facultat de Medicina, Universitat de Barcelona, Barcelona, Spain; 2 CIBER Enfermedades Respiratorias, Madrid, Spain; 3 Departament de Biologia Cel·lular, Immunologia i Neurociències, Universitat de Barcelona, Barcelona, Spain; 4 Master's and Doctoral Degree Programs in Rehabilitation Sciences, Nove de Julho University, Sao Paulo, Brazil; 5 Institut de Bioenginyeria de Catalunya, Barcelona, Spain; 6 Institut de Nanociències i Nanotecnologia, Universitat de Barcelona, Barcelona, Spain; 7 Institut d’Investigacions Biomèdiques August Pi Sunyer, Barcelona, Spain; Universidade de São Paulo, BRAZIL

## Abstract

Early morbidity and mortality in patients with Marfan syndrome (MFS) -a connective tissue disease caused by mutations in fibrillin-1 gene- are mainly caused by aorta aneurysm and rupture. However, the increase in the life expectancy of MFS patients recently achieved by reparatory surgery promotes clinical manifestations in other organs. Although some studies have reported respiratory alterations in MFS, our knowledge of how this connective tissue disease modifies lung mechanics is scarce. Hence, we assessed whether the stiffness of the whole lung and of its extracellular matrix (ECM) is affected in a well-characterized MFS mouse model (FBN1^C1039G/+^). The stiffness of the whole lung and of its ECM were measured by conventional mechanical ventilation and atomic force microscopy, respectively. We studied 5-week and 9-month old mice, whose ages are representative of early and late stages of the disease. At both ages, the lungs of MFS mice were significantly more compliant than in wild type (WT) mice. By contrast, no significant differences were found in local lung ECM stiffness. Moreover, histopathological lung evaluation showed a clear emphysematous-like pattern in MFS mice since alveolar space enlargement was significantly increased compared with WT mice. These data suggest that the mechanism explaining the increased lung compliance in MFS is not a direct consequence of reduced ECM stiffness, but an emphysema-like alteration in the 3D structural organization of the lung. Since lung alterations in MFS are almost fully manifested at an early age, it is suggested that respiratory monitoring could provide early biomarkers for diagnosis and/or follow-up of patients with the Marfan syndrome.

## Introduction

Marfan syndrome (MFS) is an autosomal dominant inherited disorder of the connective tissue, with a prevalence between 1.5 and 17.2 per 100,000 persons [1]. MFS is a systemic and severe disease with increased early morbidity and mortality whose clinical manifestations include long bone overgrowth, scoliosis, ectopia lentis and ascending aorta aneurysm development, the latter being the main death risk factor because it usually progresses to aortic dissection and rupture [1,2]. MFS is caused by mutations in the gene coding for fibrillin-1 (*FBN1*) [3–5], the major component of microfibrils in both non-elastic (ciliary zonules of the eye) and elastic (blood vessels, lung and skin) tissues [6] in which microfibrills are the scaffold for the deposition of tropoelastin [7]. Reduction or mutation of fibrillin-1 in MFS, but also in related disorders such as stiff skin and Weill-Marchesani syndromes [8], alters extracellular matrix (ECM) homeostasis, resulting in abnormal mechanical behavior.

The high-rate and high-amplitude mechanical stress that the aortic wall tissue is continuously subjected to, together with a higher structural dependence of microfibrils in aortic elastic lamellae in comparison with other enriched elastic connective tissues, would explain, at least in part, why the most life-threatening consequences of the MFS are so dramatically manifested in the aorta. The huge improvements in surgical interventions, mainly by the prophylactic repair of the dilated aortic root, as well as “preventive” pharmacological treatments developed in the last 15–20 years have resulted in a progressive reduction in the cardiovascular mortality in Marfan patients [9,10]. However, this increase in life expectancy (from 35–40 years to 65–75 years) potentially promotes the development/progression of clinical manifestations in formerly less affected tissues and organs.

Although alterations in the respiratory system are not considered a main feature of Marfan syndrome, there are several reports about these complications [11,12]. Respiratory dysfunctions include spontaneous pneumothorax, which is facilitated by widening of distal airspaces and apical blebs, emphysematous pattern tendency [13,14], chest wall deformities -such as pectum carinatum/excavatum [15] contributing to a restrictive respiratory profile [16]- and a high prevalence of obstructive sleep apnea [17]. Moreover, Corsico and co-workers [16] have very recently demonstrated that 63% of Marfan patients present alterations in lung function, as measured by spirometry, even in the absence of reported chronic respiratory symptoms. In addition to data obtained from patients, research in Marfan mice models have reported respiratory system alterations such as airspace widening [18,19] and increment in the upper airway collapsibility [20]. Altogether, these findings indicate that respiratory system evaluations in Marfan syndrome patients could be of particular interest to characterize the patient’s status.

However, how fibrillin-1 mutations and potentially resulting ECM impairments modify respiratory mechanics, specifically lung stiffness, are unexplored. Moreover, the time course of respiratory alterations in Marfan syndrome is unknown since patient studies always evaluated groups with high heterogeneity in age and disease stage [16,21]. Thus, whether pathological impairments in Marfan respiratory system are an early event and what is the temporal evolution of these alterations is unknown. Therefore, the aim of the present study was to evaluate the pulmonary mechanics, focusing in lung compliance alterations, in MFS with a time-course scope. To address this objective, we have measured *in vivo* whole lung elastance during mechanical ventilation maneuvers and we have also determined the intrinsic local stiffness of the lung extracellular matrix by atomic force microscopy (AFM), in addition of quantifying the alveolar interseptal separation, using a well-characterized mouse model of MFS (FBN1^C1039G/+^). This experimental model is based on a specific missense mutation that was found in a Marfan patient (C1039Y). Heterozygous cysteine substitutions make up the largest part of mutations found in MFS. FBN1^C1039G/+^ mice show progressive aortic aneurysm, skeletal deformities and reduced microfibrils deposition [18] similar to those found in patients.

## Methods

### Mice

Mutant C57BL/6 mice, heterozygous for a fibrillin-1 allele (Fbn1^C1039G/+^) encoding a substitution of glycine by cysteine, were used to mimic the human phenotype of classic MFS. Fbn1^C1039G/+^ mice have a normal life span and they do not die by aortic dissection [18]. C57BL/6 wild-type mice (WT) were used as controls. To analyze the effects of ageing, the study was carried out in 9-month (26.7±1.5 g and 31.2±1.2 g body-weight in MFS and WT, respectively; mean±SE) and 5-week old mice (12.6±1.1 g and 13.0±0.6 g body-weight in MFS and WT, respectively). After euthanasia at the end of the experiments, animals were genotyped conventionally by polymerase chain reaction (RT-PCR) assay of genomic DNA. Mice were housed under conventional temperature and humidity conditions in the institutional animal facility, with food and water available *ad libitum*. All experimental protocols were approved by the Ethical Committee for Animal Research of the University of Barcelona, in agreement with local and national laws and regulations.

### *In vivo* measurement of lung elastance

Respiratory mechanics was conventionally assessed during mechanical ventilation. The mouse was anesthetized by intraperitoneal injection of urethane (1.5 g/kg), tracheostomized and tightly intubated with a metallic cannula (18- and 19-gauge for 9-month and 5-week old animals, respectively). Subsequently, the mouse was placed in supine position and paralyzed with pancuronium bromide (0.2 mg/kg) by retro-orbital injection and the cannula was connected to a custom built volume-controlled ventilator (room air, 100 breaths/min). No positive end-expiratory pressure (PEEP) was applied. Tidal volume (V_T_) was set to 0.30 mL and 0.15 mL for 9-month (WT: n = 7, MFS: n = 7) and 5-week (WT: n = 8, MFS: n = 6) old mice, respectively. Ventilatory flow (V’) was recorded at the entrance of the tracheal cannula using a pneumotachograph (linear range ±20 ml/s) and a differential pressure transducer (DC001NDC4, ±2.5 cmH_2_O, Honeywell S&C). Tracheal pressure (P_tr_) was measured by connecting a transducer (176PC14HD2, ±35 cmH_2_O, Honeywell S&C) on a side port placed between the pneumotachograph and the cannula. The transducer signals were analogically low-pass filtered (Butterworth, 8 poles, 32 Hz) and sampled at a rate of 100 Hz (PCI-6036, National Instruments) through a custom monitoring and recording application (LabView). The volume signal (V) was calculated by digital integration of V’ and the P_tr_ signal was corrected by subtracting the estimated pressure drop contribution of the nonlinear pressure-flow relationship of the corresponding intubation cannula (previously calibrated).

The static (E_st_) and dynamic (E_dyn_) elastances of the total respiratory system were determined by means of the end-inspiratory airway occlusion method [22,23]. After an end-inspiratory occlusion, there is a fast initial drop in P_tr_ (ΔP_1_) from the pre-occlusion value down to an inflection point (with pressure P_i_), followed by a slow pressure decay (ΔP_2_) until a plateau pressure (P_el_) corresponding to the elastic recoil pressure of the system is reached. Whereas ΔP_1_ is associated with pressure dissipated against pulmonary resistance, ΔP_2_ reflects tissue viscoelastic properties or pendeluft. Accordingly, static elastance (E_st_) was calculated as the adjusted plateau pressure (P_el_-PEEP) recorded after 5 s of occlusion divided by V_T_, and dynamic elastance (E_dyn_) was computed by dividing the adjusted inflection point pressure (P_i_-PEEP) by V_T_. For each animal, E_st_ and E_dyn_ were obtained as the mean from two end-inspiratory occlusions, each one carried out after 1 min of continuous mechanical ventilation. After measuring total respiratory system elastances as described in the intact animal, lung E_st_ and E_dyn_ were similarly measured. To this end, the chest wall was completely opened and an external PEEP = 2 cmH_2_O was applied. E_st_ and E_dyn_ of the chest wall in each animal were computed as the difference between total respiratory system and lung elastances (for both E_st_ and E_dyn_).

### Lung decellularization

After *in vivo* elastance measurements, the mouse was euthanized by exsanguination through the abdominal aorta and the lungs and trachea were harvested. Lungs were subsequently cleaned using 1x phosphate-buffered saline (PBS) and any attached esophageal, lymphatic and connective tissues were removed. The left lung lobe of each animal was frozen and stored at -80°C until decellularization [24]. Decellularization was performed by a procedure based on repetitive freezing/thawing cycles [25] and the use of 1% sodium dodecyl sulfate (SDS) detergent. Briefly, the freezing–thawing (-80°C /20°C) was carried out four times and the lung lobe was subjected to several rinses through the bronchus using a catheter coupled to a 2.5 ml syringe: 7 washes with 2.0 ml of PBS, 5 washes with 2 ml of deionized water, and a final wash with 2 ml of SDS. A pause after each of these single washes allowed the solution to come out as lung recoiled. The lobe was kept in agitation for 48 h at room temperature in a 15 ml polystyrene conical tube with 7 ml of SDS. Then, it was again rinsed 7 times through the bronchus with 2 ml of PBS and maintained in agitation in a new conical tube with 7 ml of PBS for 48 h to remove all detergent residues. The acellular left lung scaffolds were stored at 4°C in PBS containing 2% penicillin-streptomycin solution until slice preparation.

### Measurement of elastances in *in vivo* and decellularized lungs

To assess whether potential differences in lung E_st_ and E_dyn_ between MFS and WT depend on organ decellularization we carried out a parallel run of experiments with additional mice (3-month old; WT (n = 3) and MFS (n = 4)). Using the methods previously explained, lung E_st_ and E_dyn_ were first measured *in vivo* during open chest ventilation (V_t_ = 0.20 ml, 100 bpm). Subsequently, the lungs were excised, subjected to the described decellularization protocol, tracheally canullated and mechanically ventilated (V_t_ = 0.20 ml, 100 bpm, PEEP = 2 cmH_2_O) including end-inspiratory pauses to measure E_st_ and E_dyn_ of the decellularizad whole-lung [25].

### Measurement of the local stiffness of lung extracellular matrix

Slices of lung ECM of mice of interest were obtained from the decellularized left lobe. The sample was bronchially infused with a 3:1 ratio mixture of Optimal Cutting Temperature Compound (OCT, Tissue-Tek, Sakura, Torrance, CA) and PBS, and kept at -80°C overnight. Subsequently, 12 μm-thick sections were cut using a cryostat (HM 560, CryoStar Thermo Scientific), placed on top of positively charged glass slides (Thermo Fisher Scientific, Waltham, MA), and stored at -20°C.

Extracellular lung matrix slices from 9-month old (WT and MFS, n = 4 each) and 5-week old mice (WT and MFS, n = 4 each) were allowed to warm up at room temperature for 30 min. Before atomic force microscopy (AFM) measurements, slices were rinsed several times with PBS until the OCT was completely removed. The perimeter of glass slides was outlined with a water repellent marker (Super PAP PEN, Invitrogen), keeping 1 ml of PBS pooled over the samples. Then, slides were placed on the sample holder of a custom-built AFM attached to an inverted optical microscope (TE2000; Nikon). The Young’s modulus (*E*) of the decellularized matrix samples were measured with a Si_3_N_4_ V-shape Au-coated cantilever with a four-sided blunted pyramidal tip on its apex with a semi-included effective angle (θ) of 20° with a nominal spring constant (k) of 0.03 N·m^-1^ (MLCT, Bruker, Mannheim, Germany), which was previously calibrated by the thermal tune method. 3-D piezoactuators coupled to strain gauge sensors (Physik Instrumente, Karlsruhe, Germany), allowed to place the cantilever on the region of interest with nanometric resolution and to measure the vertical displacement of the tip (z). The deflection of the cantilever (d) was measured with a quadrant photodiode (S4349, Hamamatsu, Japan). Before each slice measurement, a bare area of the coverslip was identified in order to obtain a d-z curve. Its slope was used to calibrate the relationship between the photodiode signal and the cantilever deflection. A linear calibration curve with a sharp contact point was taken as indicative of a clean and undamaged tip. The force (F) on the cantilever was computed as *F* = *k* ∙ *d*.

Local *E* of the lung ECM was measured in four different regions: pleural membrane, alveolar septum, and adventitia and media tunicae of blood vessels. Each region was assessed at three different sites. In each of these sites, five 5-μm-separated points were measured, and each measurement consisting of five force–displacement curves (F–z) (triangular ramp, 1 Hz oscillation, 5 μm peak-to-peak ramp amplitude, and a maximum indentation of ≈1000 nm). The indentation of the sample (δ) was computed as *δ* = (*z* − *z*_0_) − (*d* − *d*_0_), where z_0_ and d_0_ are the positions of the contact point. Force measurements were corrected for the hydrodynamic drag force (F_d_) on the cantilever [26] and force-displacement curves were analyzed with the ideal pyramid Hertz model

$$F=\frac{3∙E∙tan\theta }{4∙(1-v^{2})}∙\delta^{2}$$ (1)

where ν is the Poisson’s ratio (assumed to be 0.5). The ideal pyramid Hertz model can be applicable to a blunted pyramidal cantilever tip for indentations deeper than ~300 nm [27]. Eq 1 can be expressed in terms of z and d as

$$d=d_{0}+\frac{3∙E∙tan\theta }{4∙k∙(1-v^{2})}∙{\left[\left(z–z_{0})–(d–d_{0}\right)\right]}^{2}$$ (2)

*E*, z_o_ and d_o_ were computed for each force–indentation curve by non-linear least-squares fitting using a custom built software (MATLAB, The MathWorks) The fitting was performed up to a maximum indentation of 500 nm (less than 10% of the height of the sample) to avoid the effect of the underlying glass slide, as we verified by exploring indentation depths up to 1 μm in the same way as reported in previous AFM measurements on decellularized lungs [24]. For a given measurement point, *E* was computed as the average of the Young’s modulus corresponding of the five force–indentation curves. *E* for each zone in a given sample was computed as the average of the *E* values obtained for the five different points measured on that site.

The viscoelastic properties of the lung matrix, assessed by its complex shear modulus (G*) were evaluated at the alveolar septum. The tip was placed at an operating indentation (δ_0_) of 500 nm, and a small amplitude (75 nm) multifrequency oscillation composed of four sine waves (0.35, 1.15, 3.55, 11.45 Hz) was applied for 140 s. Each of these frequencies was non-sum and non-difference of the others to avoid harmonic cross-talk. G* was computed at each frequency (*f*) as:

$$G^{*}(f)=\frac{\left(1-v\right)}{\left(3∙\delta_{0}∙tan\theta \right)}∙\left[\frac{F(f)}{\delta (f)}-i∙f∙b(h)\right]$$ (3)

where F(*f*) and δ(*f*) are the frequency spectra of force and indentation, respectively. The term i·*f*·b(h), *i* being the imaginary unit, accounts for the correction of the viscous drag of the cantilever. The value of b(h) corresponding to the average thickness of the matrix sample (h = 12 μm) was used. G*(*f*) was separated into their real and imaginary parts G*(*f*) = *G*′(*f*) + *i* ∙ *G*″(*f*), where G’(*f*) and G”(*f*) represents the elastic and viscous modulus, respectively. In each sample, G* was calculated as the average of values obtained from 3 different zones of the alveolar septa. These average values were fitted in the complex plane to a linear superposition of a two power-law model:

G*(f)=A∙(i∙f)α+B∙(i∙f)34 (4)

The fit was carried out after normalizing *f* to 1 Hz. This model assumes a low-frequency regime described by a power law with a weak exponent and a high-frequency regime defined by a power law with exponent of 3/4.

### Histological processing and image analysis

Intact lungs were excised as previously described, from other 9-month old (WT and MFS; N = 6 each) and 5-week old mice (WT and MFS; n = 6 each). Each lung was fixed with 10% formaldehyde, dehydrated and embedded in paraffin. Paraffin blocks were sectioned at 5 μm thickness and histological sections were stained with hematoxylin- eosin (H-E). Images from the regions of interest were acquired using a digital camera (UI-1240SE-C-HQ, IDS) coupled to an inverted light microscope (Ti-Eclipse, Nikon, Tokyo, Japan) with 4x and 10x objectives, operated by a commercial acquisition software (uEye Cockpit, IDS, Germany), and assessed with ImageJ.

Quantitative analysis was carried out measuring interseptal air spaces. Large blood vessels and spurious structures were removed from the image to avoid confusion with enlarged alveolar spaces. Then, the image was converted into a binary-quantified image. The binary alveolar structure was next reduced to a skeletonized structure image. It was multiplied by a grid mask, which included a series of probe lines (11 vertical and 9 horizontal) equally distributed. The resulting cross-point image represented the sites where the alveolar structure intercepted the probes. Data were recorded and exported for analyses. Each mean linear intercept length (L_m_) was calculated as the quotient between the total probe length and the total number of intercepts [28–29].

Aortas were collected after euthanasia and lung excision from the same mice used for lung measurements. The aortas were sectioned immediately above the aortic valve and the proximal segment corresponding to the ascending aorta was subsequently fixed in 10% formaldehyde and embedded in paraffin. Paraffin blocks were sectioned at 5 μm thickness and stained with Verhoeff-Van Gieson to evaluate elastic fiber integrity by counting the number of fiber breaks per length.

### Assessment of ageing effects in Marfan lungs

The time course of changes in the lungs of MFS along age was completed in a parallel series of experiments where additional MFS and wild-type mice of ages covering a wide range were investigated (in addition of previously explored 5-week and 9-month): newborn (postnatal day 1), 3-month, and 6-month. In these animals, lung effective elastance E_L_ and alveolar intercept length L_m_ were measured in all animals as described previously [25]. E_L_ was not measured in newborn mice because the ventilator could not be used due to their very small lungs.

### Statistical analyses

Statistical analyses were performed with SigmaPlot (Systat Software, San Jose, CA). All values are expressed as mean ± SE. Elastances (E_st_ and E_dyn_) obtained for each age were analyzed by comparing WT and MFS groups with unpaired *t*-tests (or non-parametric Mann-Withney test when required). Differences in Young’s modulus *E* in decellularized lung regions between WT and MFS groups were analyzed by two-way analysis of variance, being lung region and animal strain/group the two factors. The complex shear modulus (G*) was analyzed using two-way analysis of variance, being frequency and strain/group the two factors. Differences between parameters of the two power law model were compared by age using two-way analysis of variance with repeated measurements and post hoc pairwise multiple comparison tests (Holm–Sidak method). L_m_ was analyzed using two-way analysis of variance (age and strain/group) and post hoc pairwise multiple comparison tests (Holm–Sidak method). In all cases, tests with p < 0.05 were considered statistically significant.

## Results

### Lung elastance and extracellular matrix stiffness in young and adult Marfan mice

**Fig 1:**
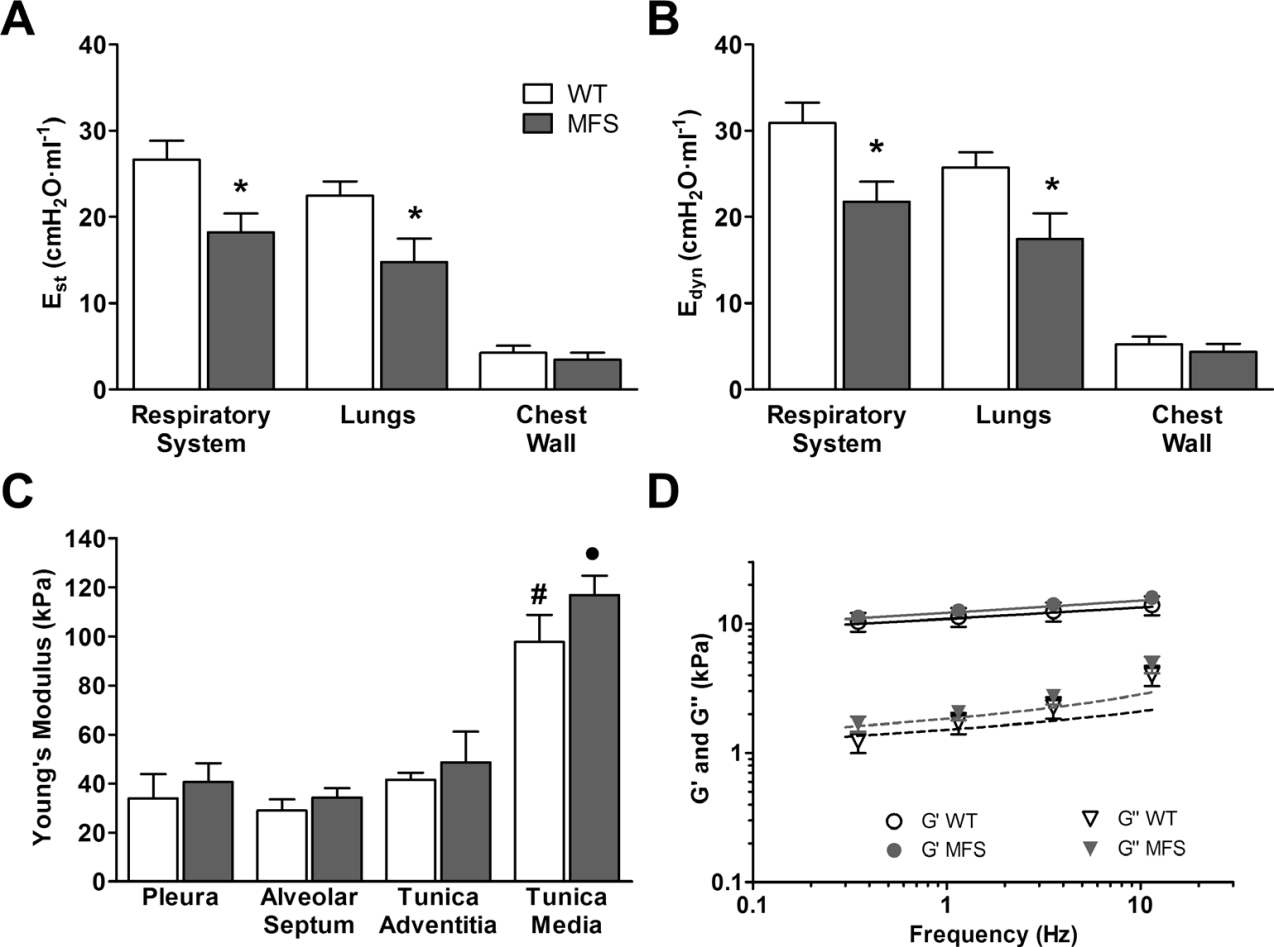
Mechanics in 9-month old mice. (A) Static (E_st_) and (B) dynamic (E_dyn_) elastances in WT (white column) and Marfan mice (grey column) determined by the end-inspiratory airway occlusion method. *: p < 0.05 when comparing WT and Marfan. (C) Young’s modulus (E) measured at different regions of the lung ECM in WT (white column) and Marfan mice (grey column). #: p < 0.05, and •: p < 0.05 when comparing different regions in WT and Marfan, respectively. (D) Complex shear modulus (G*) measured at the alveolar septum of decellularized lung matrix in WT (open symbols) and MFS (closed symbols) mice. Circles represent the real part (storage modulus, G’) and triangles represent the imaginary part (loss modulus, G”) of G*. Solid and dashed lines represent fits of the two power law model to G’ and G”, respectively (WT black and MFS grey). Data are mean ± SE.

Lung elastance, which indicates the amount of pressure required to insufflate a given air volume in the lung, is the more direct index to assess how easy or difficult it is to ventilate the organ. Specifically, lung elastance–the reciprocal of lung compliance- quantifies lung stiffness. The total respiratory system of Marfan mice at the adult age of 9-month presented significantly lower static elastance (E_st_) values (were more compliant) than those of wild-type (WT) animals (Fig 1A). Elastance measurements obtained after opening the mice chest wall provided values corresponding to lung stiffness. Lung E_st_ in MFS mice were more compliant than WT lungs: on average E_st_ was 34% lower in MFS mice. This decrease accounted for the significant variation in the total respiratory system E_st_, since chest wall E_st_ -calculated as the difference between respiratory system E_st_ and lung E_st_- was the same in both mice groups (Fig 1A). Differences in dynamic elastances (E_dyn_) (Fig 1B) between groups were highly similar to those found in static elastances. E_dyn_ values were slightly higher than E_st_ ones (Fig 1A and 1B) as expected for a viscoelastic system [30]. In fact, E_dyn_/E_st_ ratio was close in WT (1.15±0.01) and Marfan mice (1.19±0.02).

Whereas lung elastance provides an index indicating how difficult it is to inflate a whole lung, i.e. it is a bulk macroscopic measurement, the Young’s modulus *E* of the lung scaffold is an index of how stiff the ECM is at a local level (within submicron dimensions). Hence, to assess whether the observed changes between WT and MFS in whole-lung compliance could be attributed to lung ECM stiffness, *E* was measured with AFM by locally testing the deformability of the lung matrix at specific sites. As expected [24,31], the Young’s modulus *E* of the ECM significantly varied among different lung regions (p<0.05) (Fig 1C). Specifically, there was no regional difference in the elastic properties of the lung tissue, with the exception that the tunica media was stiffer than the other regions (Fig 1C). Remarkably, in contrast with *in vivo* lung elastances (Fig 1A and 1B), no significant differences in the stiffness *E* of the lung ECM were found when comparing MFS and WT animals (Fig 1C). In addition of *E* (which indicates static stiffness) AFM also allowed to measure the viscoelastic (i.e. dynamic) properties of the lung ECM by applying different frequencies of local oscillation to compute the complex shear modulus G*. The complex shear modulus of MFS and WT decellularized lung samples showed the same weak frequency dependence which was very well fitted with the two power-law model (Fig 1D). No significant differences were found when comparing the model parameter values (A, B, α) between MFS and WT (Table 1).

**Table 1:** Viscoelastic parameters of the lung ECM in WT and Marfan mice.

Age	9-month		5-week	
**Type**	*Wild Type*	*Marfan*	*Wild type*	*Marfan*
**A (kPa)**	9.40 (±1.30)	10.34 (±0.79)	6.64 (±1.02)*	9.63 (±2.14)
**B (kPa)**	0.016 (±0.010)	0.036 (±0.022)	0.030 (±0.003)	0.066 (±0.018)
**α**	0.084 (±0.010)	0.089 (±0.011)	0.060 (±0.011)	0.072 (±0.015)

Parameters of the two power-law model G*(f)=A∙(i∙f)α+B∙(i∙f)34 fitted to data measured in alveolar septa of decellularized lung matrix. Data are mean ± SE. *: p< 0.05 for 5-week vs 9-month old wild type mice.

**Fig 2:**
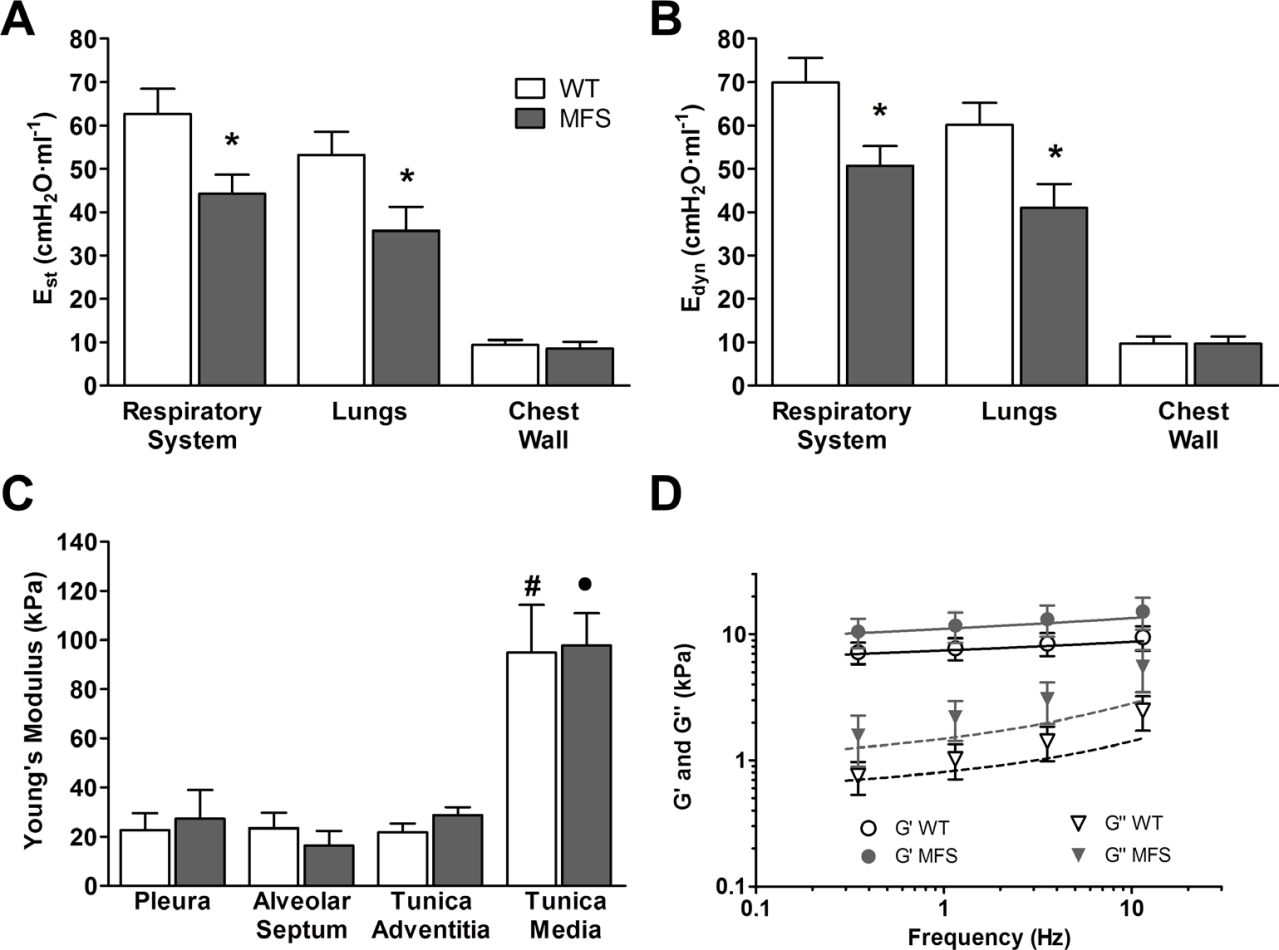
Mechanics in 5-week old mice. (A) Static (E_st_) and (B) dynamic (E_dyn_) elastances in WT (white column) and Marfan mice (grey column) determined by the end-inspiratory airway occlusion method. (C) Young’s modulus (E) measured at different regions of the lung ECM in WT (white column) and Marfan mice (grey column). (D) Complex shear modulus (G*) measured at the alveolar septum of decellularized lung matrix in WT (open symbols) and MFS (closed symbols) mice. Circles represent the real part (storage modulus, G’) and triangles represent the imaginary part (loss modulus, G”) of G*. Solid and dashed lines represent fits of the two power law model to G’ and G”, respectively (WT black and MFS grey). Data are mean ± SE. *: p < 0.05.

In order to compare the putative evolution of biomechanical lung alterations, we also measured the same aforementioned mechanical parameters in lungs of 5-week old mice as a representative age for evaluating early events occurring in MFS. Interestingly, when comparing whole-lung elastance, ECM stiffness (*E*) and ECM viscoelasticity (G*) in 5-week old MFS and WT mice, we found the same differences as those found in 9-month old adult mice (Fig 2, Table 1).

### Comparison of E_st_ and E_dyn_ in *in vivo* and decellularized lungs

**Fig 3:**
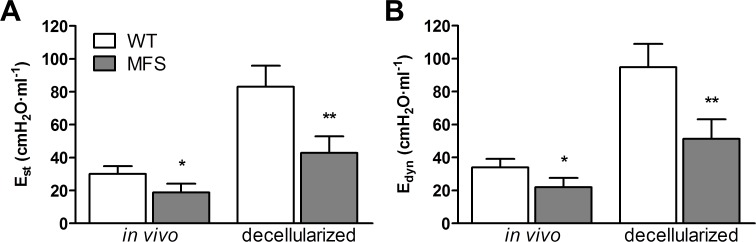
Lung elastances before and after decellularization. (A) Static (E_st_) and (B) dynamic (E_dyn_) elastances in WT (white column) and Marfan mice (MFS; grey column) determined by the end-inspiratory airway occlusion method in vivo and in the acellular lung scaffolds after organ decellularization. Data are mean ± SE. *: p < 0.05; **: p < 0.01.

Fig 3 shows whole lung elastances E_st_ and E_dyn_ of the same mice before (in vivo) and after decellularization for both MFS and WT mice. As expected from the results previously shown in Figs 1 and 2, in vivo lung elastances were significantly (p<0.05) lower in MFS than in WT mice. Specifically, *in vivo* E_st_ and E_dyn_ were 37% and 35% lower in MFS than in WT animals. As it is well known [32], lung elastances after organ decellularization were considerably higher than *in vivo*. The interesting result of this series of experiments was that differences between MFS and WT lungs persisted when decellularized: E_st_ and E_dyn_ were 51% and 45% lower in MFS than in WT animals. These data indicate that MFS and WT mice have different in lung stiffness when the mechanics of the only 3-D extracellular matrix of the lung is measured.

### Histological lung alterations in young and adult Marfan mice

**Fig 4:**
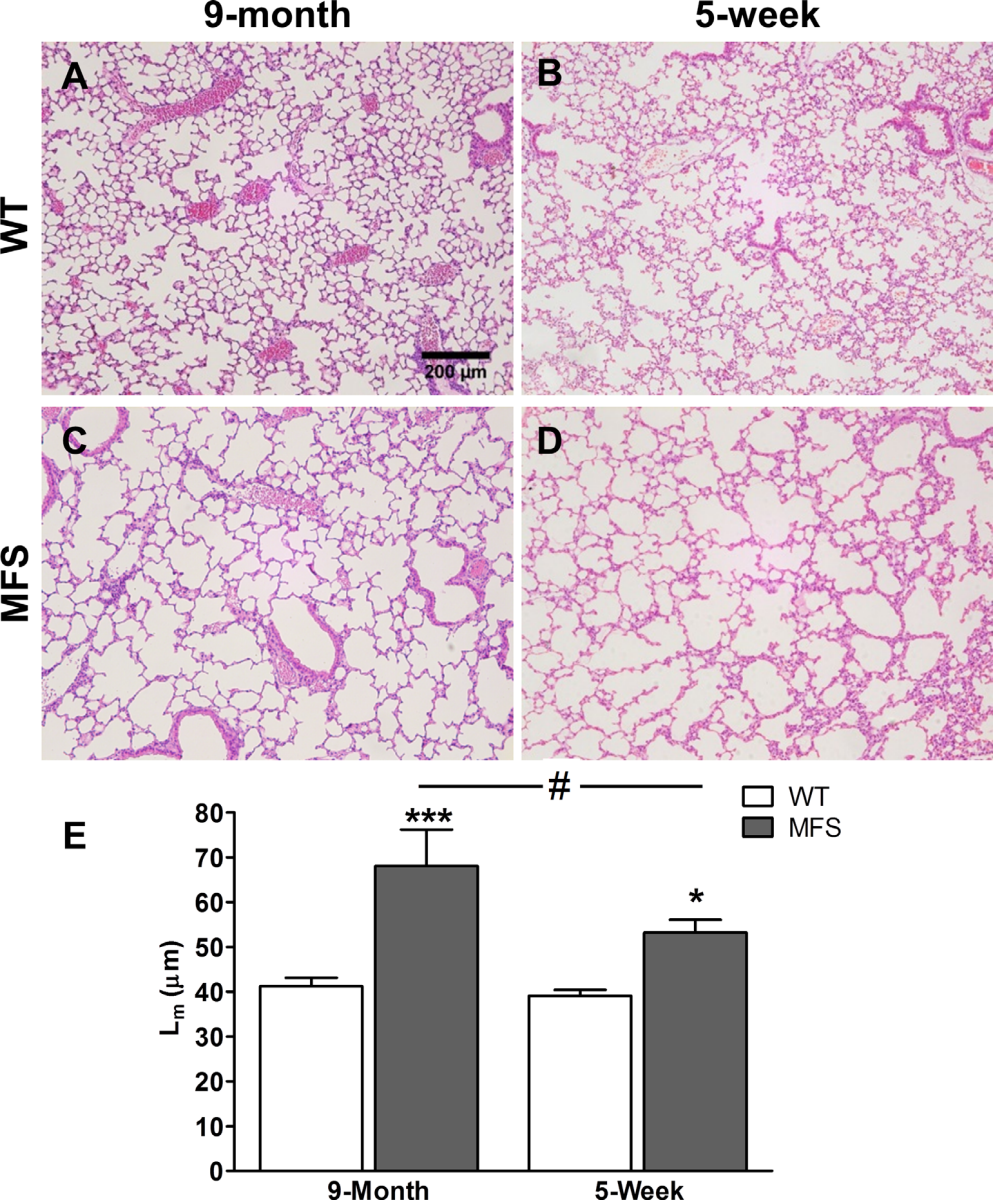
Histological analysis of mouse lungs. Representative H&E stained images of lung sections from (A) 9-month and (B) 5-week old wild-type (WT), and (C) 9-month and (D) 5-week old Marfan (MFS) mice. Images acquired at 100x magnification and scale bar represents 200μm. (E) Mean linear intercept (L_m_) of alveolar septa. Data are mean ± SE. * p < 0.05; *** p <0.001 compared with their respective WT and # p < 0.05 between different ages in Marfan mice.

To interpret the observed biomechanical differences, we also evaluated lung histological differences between MFS and WT mice groups. Clear structural alterations were observed in the lungs of 9 month-age Marfan mice in comparison with same age WT animals: MFS mice showed a clear pattern of large emphysematous-appearing alveolar spaces (Fig 4C). Interestingly, highly similar histological alterations were also clearly observed in 5-week old Marfan mice (Fig 4D). Quantitative analysis of histological sections showed that the mean linear intercept (L_m_), representing the alveolar size, was 65% greater in MFS than in WT mice at 9-month age and 36% higher in MFS versus WT in 5-week young mice (Fig 4E). Remarkably, this Fig also shows that no significant changes in L_m_ were found when assessing the effect of age in WT mice.

### Time course of mechanical and histological alterations in Marfan mice

**Fig 5:**
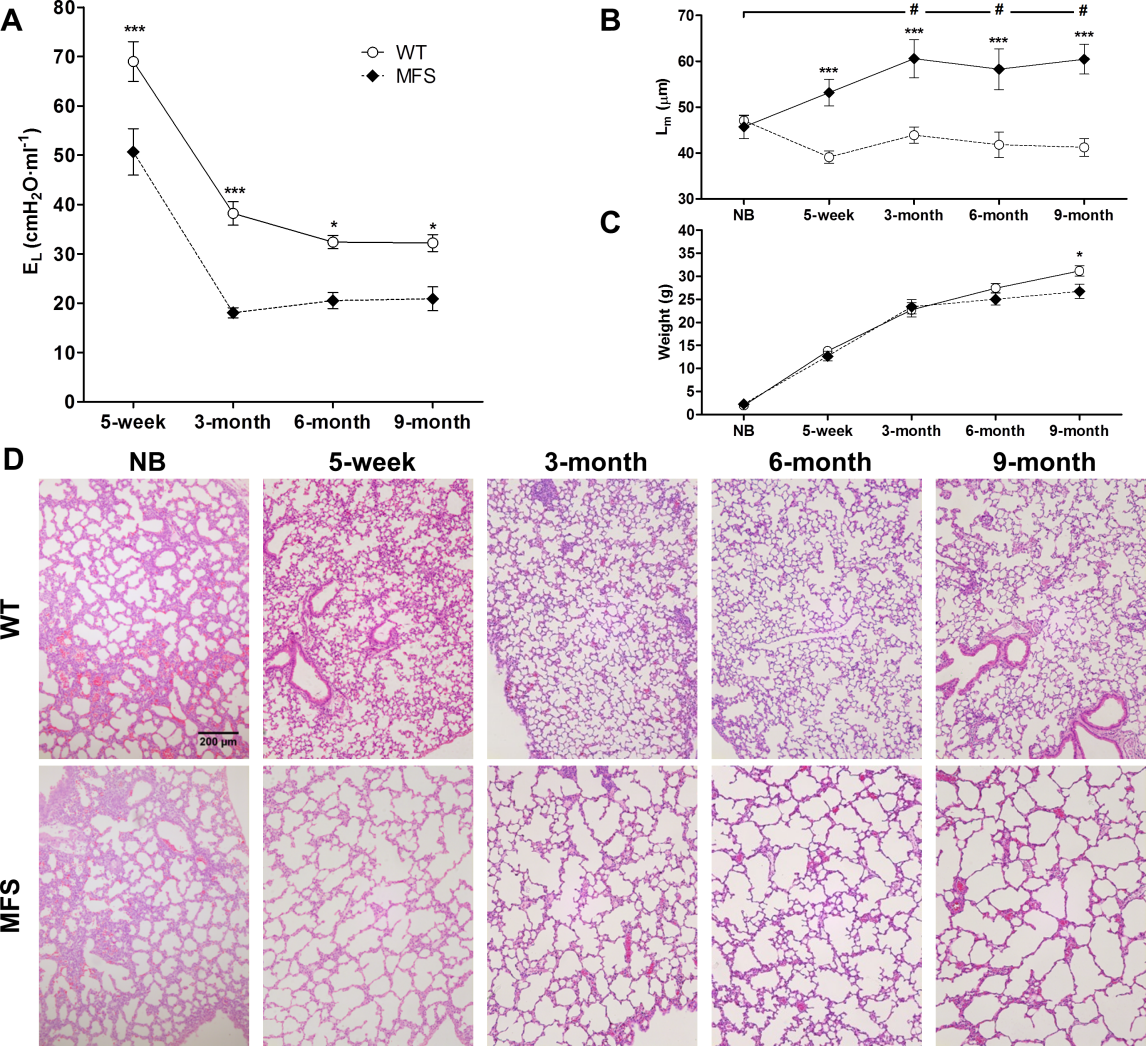
Airspace enlargement evolution in WT and MFS mice. (A) Effective lung elastance (E_L_) and (B) mean linear interecept (L_m_) of alveolar septa in mice of different ages: from newborn (NB) up to 9-month old in wild-type (WT; open circles) and Marfan (MFS; closed black circles) mice. (C) Body weight along age in MFS and WT mice. (D) Panel illustrates examples of H&E stained lung sections. Images acquired at 100x magnification and scale bar represents 200μm. Data are mean ± SE. ***: p<0.001 between WT and Marfan animals and #: p<0.05 respective to new born mice in Marfan animals.

**Fig 6:**
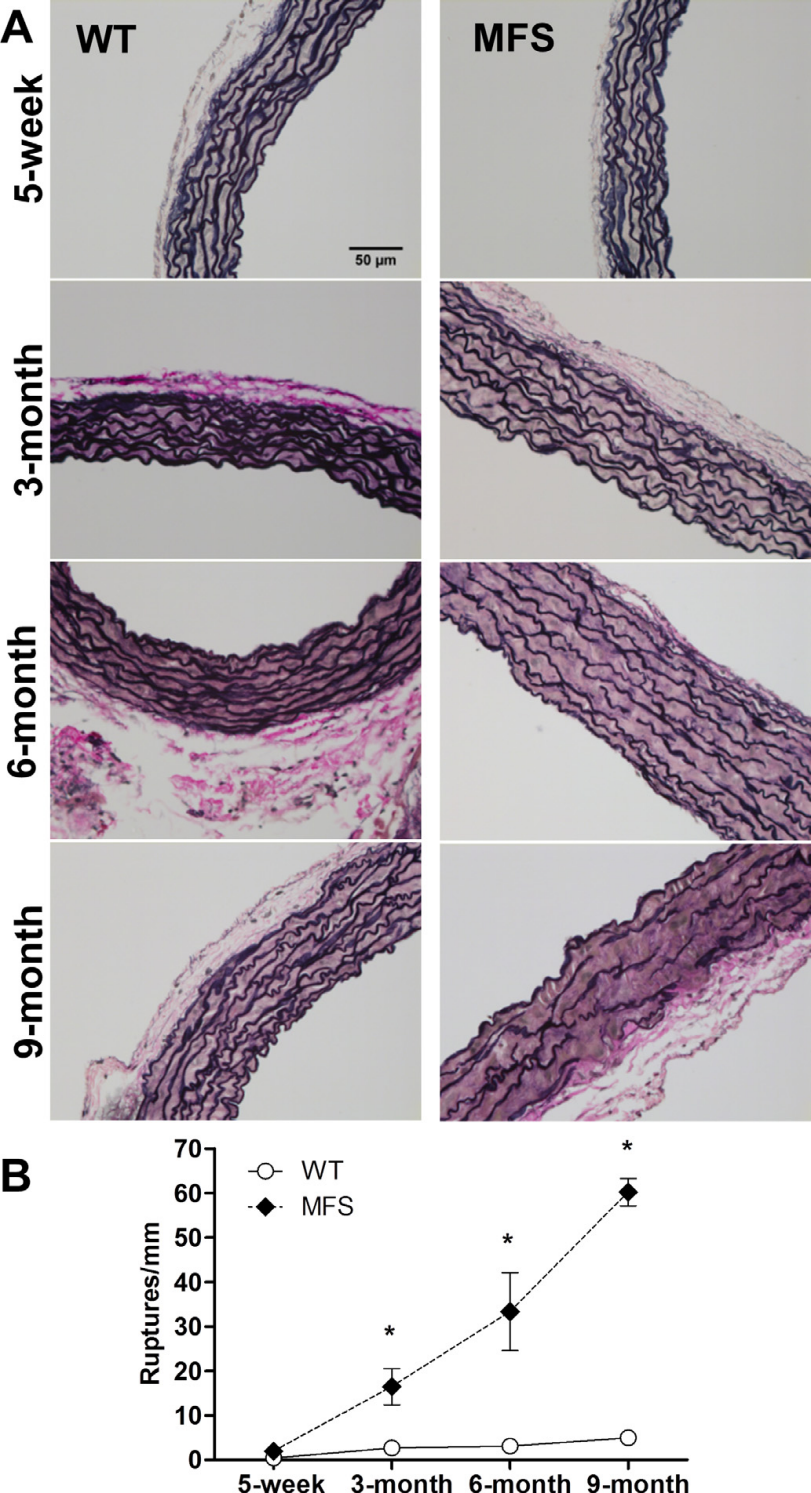
Tunica media fibers integrity in WT and MFS mice aortas. (A) Representative images from ascending aortas in WT and Marfan (MFS) mice processed and evaluated for elastic tunica media fibers integrity. Histological sections were stained with Verhoeff-Van Gieson. Images acquired at 40x magnification and scale bar represents 50μm.(B) Elastic fiber ruptures in the tunica media of the ascending aorta. Data are mean ± SE. *: p<0.05 between WT and Marfan animals

To characterize the time course of alterations in lung elastance and structure, we also studied newborn (n = 4), 3-month (n = 6) and 6-month (n = 6) old animals. Fig 5 shows the time course of effective lung elastance (E_L_) and mean linear intercept (L_m_) and body weight when data measured from all the investigated ages are represented together. Besides the already mentioned higher values of E_L_ in 5-week all mice caused by body size, Fig 5A shows that E_L_ scarcely changed from the age of 3-month to 9-month in both MFS and healthy mice. Moreover, whereas L_m_ was almost invariant over the wide range of ages in WT mice, in MFS mice the emphysematous-like structure of the lung significantly increased from newborn -with a value virtually the same than in newborn WT- to the age of 3-month old and then L_m_ remained almost constant until the age of 9-month old (Fig 5B and 5D). Body weight increased with age similarly in WT and MFS, with only significant difference at age 9-month (MFS lower body weight than WT; Fig 5C). Interestingly, the time progression of lung structural impairments in MFS mice was distinct than aortic structural injuries in the same animals, in which the elastic fiber ruptures increased progressively along the age span investigated (Fig 6).  

## Discussion

To our knowledge, this is the first study focused on assessing the elastic mechanical properties of the respiratory system in MFS. Using a mouse model of this disease we found that, *in vivo*, the total respiratory system was significantly more compliant in Marfan mice as compared with controls. This higher respiratory compliance was caused by changes exclusively in the lung since there were no differences in the elastic properties of the chest wall. The lack of contribution of the chest wall on changes in the elastic properties of total respiratory system compliance in Marfan mice is noticeable according to patient’s data [15]. When further investigating whether the observed increase in lung compliance in the Marfan syndrome could be attributed to a softening of the ECM of the lung by measuring the local mechanics of the decellularized scaffold, we found no differences between the Marfan and healthy mice. Moreover, Marfan mice exhibited a clear emphysematous histopathological pattern with larger alveolar interseptal distances. Interestingly, all these results were the same in 5-week and 9-month old mice, which, translated to humans, would range from childhood-teenagers to mature adults. A more detailed time progression evaluation revealed that the emphysematous pattern rapidly aggravated from newborn mice to 3-month old and then did not progress until mature adulthood. Accordingly, the mechanical properties of the lung in MFS would be already hampered at young ages.

This study was developed by an experimental approach combining different methodologies in an experimental Marfan mouse model (C1039G). This model mimics phenotypic aspects of human MFS, like skeletal alterations and progressive impairments of aortic wall, resulting from a cysteine substitution in fibrillin-1, a usual mutation in MFS patients [18]. First, we assessed the *in vivo* mechanical properties of the respiratory system under physiological conditions of normal ventilation by means of a technique widely used both in animals and patients [22,33,34]. Combination of closed- and open-chest measurements allowed us to separately quantify lung and chest wall elastances. Second, we measured the local stiffness of the ECM by AFM on decellularized lungs, a technique that has been recently employed in a variety of settings to assess the micromechanics of tissues and organ scaffolds [35–37]. Although measuring the local stiffness of lung ECM by AFM involved sample processing (decellularization and slice sectioning) that could slightly affect the results [24], the technique employed in this work preserves the architecture of the lung scaffold (S1 Fig) and was previously able to detect a wide range of lung ECM stiffness changes caused by ageing and fibrosis [31]. Therefore, it is expected that the lack of difference in local lung stiffness measured by AFM when comparing MFS and WT is not masked by the experimental process. The fact that differences in lung elastances (E_st_ and E_dyn_) between MFS and WT mice persisted when measured by end-inspiratory occlusions in decellularized lungs strongly supports the notion that the 3-D structure plays a fundamental role in explaining why lungs in MFS mice are softer than in control animals. Third, we used conventional histological analysis to quantify the interseptal distance by means of a previously described index L_m_ [28,29]. Interestingly, the two types of mechanical measurements (whole organ elastance and ECM stiffness) were always carried out each in the same lung specimen, reducing in this manner biological variability.

The results of whole lung elastances (E_dyn_, E_st_), ECM local mechanics (*E*, G*) and interseptal distance (L_m_) obtained in WT mice were fully consistent with the expected values. At the whole organ level mechanics, dynamic (25.7±1.8 cmH_2_O·ml^-1^) and static lung elastances (22.4±1.7 cmH_2_O·ml^-1^) in adult (9-month old) WT mice were in accordance with the literature for E_dyn_ (≈24.5–27.0 cmH_2_O·ml^-1^) and E_st_ (≈20.0–23.0 cmH_2_O·ml^-1^) [38]. The higher elastance values we found in young 5-week WT old mice (60.2±5.1 cmH_2_O·ml^-1^ and 53.3±5.3 cmH_2_O·ml^-1^, for E_dyn_ and E_st_, respectively) were expected from previous data in the literature. Indeed, our data are fully consistent with reported values of 62–70 cmH_2_O·ml^-1^ for young mice [39,40]. Changes in lung compliance induced by age (5-week vs 9-month) should be attributed both to considerable difference in body size and effects induced by age *per se* [41]. When we evaluated the local stiffness of the lung ECM of young and adult WT mice, we found a local heterogeneity like that previously reported in decellularized lungs in rats and mice [31,42]. In particular, in adult WT mice (9-month) the *E* values obtained in the pleura, septum, tunicae adventitia and media were highly similar/equivalent to those previously reported [31]. Moreover, when considering the viscoelasticity of the decellularized alveolar septum assessed by fitting G*(*f*) data to a descriptive model, parameters in 9-month old WT mice (Table 1) are fully comparable with those reported in adult healthy rats (A = 5.59±3.39 kPa, α = 0.050±0.009 and B = 0.045±0.032 kPa; [42]. Consistently with previous data obtained in normal acellular rodent lungs [31], no significant age-dependent effects were found in the static (*E*) and viscoelastic (G*) local properties of the ECM, despite 5-week old mice lungs being slightly softer than 9-month old mice lungs. Moreover, interseptal linear distance L_m_ in WT mice (41.2±1.9 and 39.1±1.3 μm in 9-month and 5-week old mice, respectively) were highly similar to those previously reported (35–42 μm) [43,44], being consistent with small (<10%) alveolar size changes from juvenile (2-month old) to senescent (20-month old) mice in two inbred strains [41].

The elastic properties of the whole lung are of most practical importance for ventilation since they are a main component in determining the inspiratory muscle effort required to breathe. Regardless of animal age, our data clearly showed that *in vivo* lung elastances (E_st_ and E_dyn_) were markedly reduced in Marfan as compared with WT mice (Figs 1, 2 and 5A). The observation that the E_dyn_/E_st_ ratio was highly similar between WT and Marfan mice regardless of their age (1.15±0.01 and 1.19±0.02 for WT and MFS, 9-month, and 1.14±0.03 and 1.16±0.02 for WT and MFS, 5-week old results) suggests that fibrillin-1 mutation mainly increases lung static compliance and that, by contrast, whole lung viscoelasticity and inhomogeneities (both determining E_dyn_/E_st_) are almost unaffected. Unlike the results found when considering *in vivo* whole lung, the static (*E*) and dynamic (G*) values of stiffness of the decellularized lung ECM did not show significant differences when comparing Marfan and WT mice. In fact, we observed a slight trend to a stiffer ECM in Marfan mice, which is in accordance with data reported in the aortic wall [18,45]. Altogether, results obtained in decellularized lungs indicate that despite the presence of mutated fibrillin-1, viscoelastic properties of the Marfan lung ECM was no different from that in WT mice. Moreover, comparing L_m_ in both mice groups indicated that the interseptal distance did not vary either with the age in the WT mice and that this variable–which is an index of emphysematous pattern- was clearly increased in MFS, being more markedly in adult (9-month old) than in young (5-week old) mice. Interestingly, the fact that lung structure and mechanics is already affected at the age of 5-week is similar to data reported on early alterations in mitral valve leaflets [46,47] and carotid arteries in fibrillin-1 deficient mice [48].

The elastic properties of the lung are determined by the stiffness of the parenchymal tissue and its structural 3D assembly, which can be differently impaired according to the disease [49,50]. For instance, in lung fibrosis the abnormal accumulation of collagen results in a stiffer ECM [32,51,52] and hence less compliant lungs, whereas in lung emphysema the cause of increased pulmonary compliance is an abnormal augmentation in the separation of alveolar septa caused by parenchymal tissue destruction [44,53]. It could be postulated that the fibrillin-1 mutation in our experimental mice model results in the alteration of the lung ECM because this glycoprotein is a microfibril constituent that plays a role as a scaffold for tropoelastin and, therefore in elastic fiber formation and organization [54]. However, we did not find alterations in the nanomechanics of Marfan mice lungs. Our observations are then more in accordance with those reported in which the AFM-measured Young’s modulus of single elastic fibers was not significantly affected by the absence or presence of fibrillin–microfibrils in single vertebrate elastic fibers [55]. It seems more plausible that the increase in lung compliance is primary caused by the fact that fibrillin-1 mutation reduces the lung matrix resistance to deformations [56], which in turn results in the destruction of alveolar walls, similarly as in elastase-induced lung emphysema [57].

The lung emphysema pattern was previously demonstrated in two distinct mouse models of Marfan syndrome [19,45]. These two MFS models (Fbn1^mgΔ/mgΔ^ and Fbn1^mgR/mgR^) and our model (Fbn1^C1039G/+^) present different kinds of fibrillin-1 mutations, covering a wide range of MFS phenotypes spectrum: from extremely severe (death at postnatal day 10), intermediate (survival until 6–9 month old) and mild (survival > 1 year), respectively. Neptune and co-workers [19] showed that both models, and even Fbn1^mgΔ/ +^ littermates -undistinguished at aortic level from WT and with normal lifespan- presented graded increase in distal airspace of lungs at different ages. Moreover, Dyhdalo and co-workers [13] histologically studying lung tissue specimens from a non-specific group of Marfan patients, showed the presence of a consistent pattern of emphysema in all samples. Altogether, our results and previous published reports suggest that the frequency of lung emphysema pattern in MFS could be higher than the currently thought. Early lung damage in MFS was previously suggested in cases reporting emphysema-like pattern in very young children with MFS [58,59] and at 9 days of postnatal life in a Fbn1^mgΔ/ mgΔ^ mouse model [19]. However, our work is the first performing a systematic study of histological lung damage and mechanical alterations from newborn till adulthood in the mildest mouse model showing that lung impairment is indeed an early onset in this genetic disease. Interestingly, we also showed that the alveolar ruptures are not presented in newborn lungs, but at 5-week lungs, suggesting that the structural lung impairment could be initiated by the cyclic stress of breathing. However, future research focusing on alveolar morphology and structure would help in understanding whether it is developmental emphysema or a consequence of the destruction of elastic tissue because of repeated stretching.

From a clinical viewpoint, the results obtained in the Marfan mouse model indicating early changes in the structural and mechanical properties of the lung points towards further equivalent research in patients with this genetic disease. Specifically, our data suggest that follow-up of the lung status from early ages could provide interesting biomarkers of how the consequences of the syndrome evolve in the respiratory system. In this context and interestingly, Marfan mice lung structural impairments clearly occurred earlier than aorta remodeling, a typical marker of aorta injury in MFS that progressively accompanies ascending aortic dilatation, one of the two main diagnostic criteria [1,60]. This finding strongly suggests that evaluation of patient lung status could be useful in MFS follow-up. Such an assessment could be routinely carried out by techniques commonly employed in respiratory medicine. Whereas direct measurement of lung compliance in patients requires a relatively cumbersome technique, other indices easily measured in the lung function lab (lung volumes and diffusion capacity) provide clues to detect alteration in compliance and emphysematous pattern. Moreover, imaging the lung by computed tomography scanning is particularly suited for identifying emphysema-like alterations in lung structure.

In conclusion, heterozygous mutation of fibrillin-1 gene, which is the causative hallmark of MFS, leads to an increase in lung compliance compatible with an emphysematous-like remodeling of the alveolar structure. Remarkably, these alterations are almost fully manifested at an early age. Besides the obvious limitations derived from using a specific mutant Marfan mouse model, the novel results reported in this work are useful to interpret the respiratory alterations observed in Marfan patients and opens new questions for further investigation. Better understanding the respiratory consequences of this genetic disease of the connective tissue will be more relevant with the expected increase in the presentation of lung dysfunctions as patients’ survival is improved thanks to successful therapies to reduce their early cardiovascular complications.

## Supporting Information

S1 Fig Decellularized lung slice probed by atomic force microscopy (AFM). Phase contrast image of a 12 μm-thick section showing the AFM cantilever. Scale bar = 50 μm.
(PDF)
