# Supplementary figures and images for: Early Impairment of Lung Mechanics in a Murine Model of Marfan Syndrome
Source: PLoS One. 2016 Mar 22;11(3):e0152124. doi: 10.1371/journal.pone.0152124 (PMC4803219; doi:10.1371/journal.pone.0152124)

***S1 Figure***

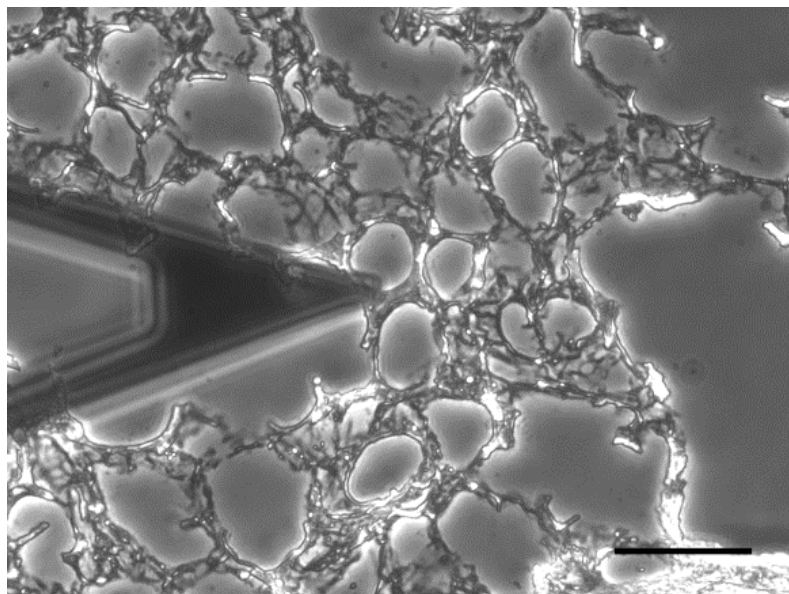

Supplement: S1 Fig — Phase contrast image of a 12 μm-thick section showing the AFM cantilever. Scale bar = 50 μm. (PDF) [file pone.0152124.s001.pdf]
